# Imaging peripheral nerve micro-anatomy with MUSE, 2D and 3D approaches

**DOI:** 10.1038/s41598-022-14166-1

**Published:** 2022-06-17

**Authors:** Chaitanya Kolluru, Austin Todd, Aniruddha R. Upadhye, Yehe Liu, Mikhail Y. Berezin, Farzad Fereidouni, Richard M. Levenson, Yanming Wang, Andrew J. Shoffstall, Michael W. Jenkins, David L. Wilson

**Affiliations:** 1 Department of Biomedical Engineering, Case Western Reserve University, Cleveland, OH 44106 USA; 2 University of Texas Health Science Center at San Antonio, San Antonio, TX 78229 USA; 3 APT Center, Louis Stokes Cleveland VA Medical Center, Cleveland, OH 44106 USA; 4 Department of Radiology, Washington University in St. Louis, St. Louis, MO 63110 USA; 5 Department of Pathology and Laboratory Medicine, UC Davis Health, Sacramento, CA 95817 USA; 6 Department of Radiology, Case Western Reserve University, Cleveland, OH 44106 USA; 7 Department of Pediatrics, Case Western Reserve University, Cleveland, OH 44106 USA

**Keywords:** Peripheral nervous system, Biomedical engineering, Microscopy

## Abstract

Understanding peripheral nerve micro-anatomy can assist in the development of safe and effective neuromodulation devices. However, current approaches for imaging nerve morphology at the fiber level are either cumbersome, require substantial instrumentation, have a limited volume of view, or are limited in resolution/contrast. We present alternative methods based on MUSE (Microscopy with Ultraviolet Surface Excitation) imaging to investigate peripheral nerve morphology, both in 2D and 3D. For 2D imaging, fixed samples are imaged on a conventional MUSE system either label free (via auto-fluorescence) or after staining with fluorescent dyes. This method provides a simple and rapid technique to visualize myelinated nerve fibers at specific locations along the length of the nerve and perform measurements of fiber morphology (e.g., axon diameter and g-ratio). For 3D imaging, a whole-mount staining and MUSE block-face imaging method is developed that can be used to characterize peripheral nerve micro-anatomy and improve the accuracy of computational models in neuromodulation. Images of rat sciatic and human cadaver tibial nerves are presented, illustrating the applicability of the method in different preclinical models.

## Introduction

Peripheral nerves act as bidirectional pathways, forming communication networks between the central nervous system and other parts of the body. Owing to their key role in the nervous system and relative ease of accessibility, electrical stimulation of these nerves has shown great promise in treating a wide range of neurological conditions and injuries^1,2^. However, current understanding of peripheral nerve micro-anatomy is limited, resulting in design challenges of neural interfaces. For instance, anatomical features of the nerve (e.g., fascicle diameter, perineural sheath thickness, fiber arrangement within fascicles, myelin sheath thickness, and proximity to extraneural electrodes) are known to significantly influence a fiber’s response to stimulation^3–5^. As researchers continue to improve neural interface designs, it is also important to map nerve micro-anatomy to achieve the desired stimulation patterns and maximize therapeutic efficacy. This manuscript builds on a novel imaging tool to address this problem.

Currently available imaging methods are limited in their ability to image peripheral nerve micro-anatomy across long lengths. The gold standard method of resin-based histology enables automated analysis of nerve morphology on thin sections ^6^; however, methods to reconstruct a 3D volume from serial sections have proven to be challenging^7^. Imaging with micro-CT can provide volumetric datasets across longer distances but resolution and contrast are limited to visualizing fascicle bundles^8^. Recently, diffusion MRI methods have been applied to characterize fiber tracts within peripheral nerves. Although this method has the benefit of imaging in vivo, imaging at a high resolution results in a low signal-to-noise ratio (SNR) and long imaging times^9^. In the optical microscopy domain, label-free methods such as Coherent anti-Stokes Raman scattering microscopy utilize physical properties of lipid and myelin sheaths to generate contrast^10^; however, such systems require expensive laser sources and are limited in imaging depth. Developments in light-sheet microscopy can be utilized to image large samples, but these methods require the use of tissue clearing reagents which can distort the tissue, making it not well suited for morphometric analyses^11^. In this regard, a comprehensive validation of the light-sheet approach in human cadaver nerve tissues is required. Additionally, the working distance of the objective limits the achievable depth, which can be a concern when imaging large human nerves. Thus, developing an imaging tool that is easy to implement, has sufficient resolution and contrast to visualize nerve micro-anatomy over a large field of view can address a critical need in peripheral nerve imaging.

We pursue a slide-free, technically simple, and cost-effective microscopy technique, Microscopy with Ultraviolet Surface Excitation (MUSE)^12,13^ to image nerve micro-anatomy. Briefly, MUSE imaging uses ultraviolet (UV) excitation at 280 nm and captures fluorescence images of the tissue surface using a color camera. The MUSE system provides several advantages. First, the limited tissue penetration depth of UV light at 280 nm provides optical sectioning by confining fluorescence excitation to the tissue surface, reducing subsurface fluorescence that can degrade effective resolution and contrast. Second, a wide range of commonly used MUSE dyes are fluorescent in the visible range, allowing image acquisition with a color camera. Third, UV light in the appropriate spectral ranges can be generated with inexpensive LED sources. As UV light below 300 nm is absorbed by glass, the microscope optics intrinsically block stray excitation light, thus eliminating the need for specific excitation and emission filter sets. Finally, the process of creating images of the tissue surface with this technique is almost instantaneous, providing a significant time savings compared to conventional histology. Previous reports have utilized this method for imaging surgical specimens of breast and skin cancer using bench-top systems^14,15^ and in the implementation of a smartphone-based portable fluorescence microscope^16^. Depending on which structures are being visualized, tissue auto-fluorescence may provide sufficient contrast with suitable illumination patterns^17^; in other cases, the addition of fluorescent dyes or labeling agents may be necessary.

In order to extend MUSE to image a 3D volume, we utilize a block-face imaging approach. A number of block-face imaging techniques have been previously reported^18–25^, but are often limited in resolution due to subsurface fluorescence. Some approaches have applied deconvolution-based techniques^18,26^ to remove out-of-plane blurring; these methods can have limited success and prove to be computationally intensive when applied to large datasets. Alternatively, few approaches have used confocal^19^ or multi-photon^20^ imaging to achieve optical sectioning on the block face, but these systems are complex and costly. To address this issue, we build on earlier reports^27^ and utilize the advantages of MUSE microscopy described previously for imaging the block face. A resin-based embedding medium is used to create tissue blocks since it is known to preserve morphology better than paraffin^28^.

In this report, we will present methods to image peripheral nerve samples with MUSE. We determined tissue preparation methods utilizing either fixative auto-fluorescence or stain with suitable dyes. We found that resolution and field of view were sufficient to image micro-anatomical features of interest (e.g., fascicles, connective sheaths, and individual nerve fibers) in rat and human cadaver samples. We utilized both 2D and 3D MUSE approaches to determine their characteristics for nerve imaging. We are specifically interested in the capability to use 3D MUSE over extended lengths of nerve tissue.

## Results

### 2D-MUSE imaging

**Figure 1:**
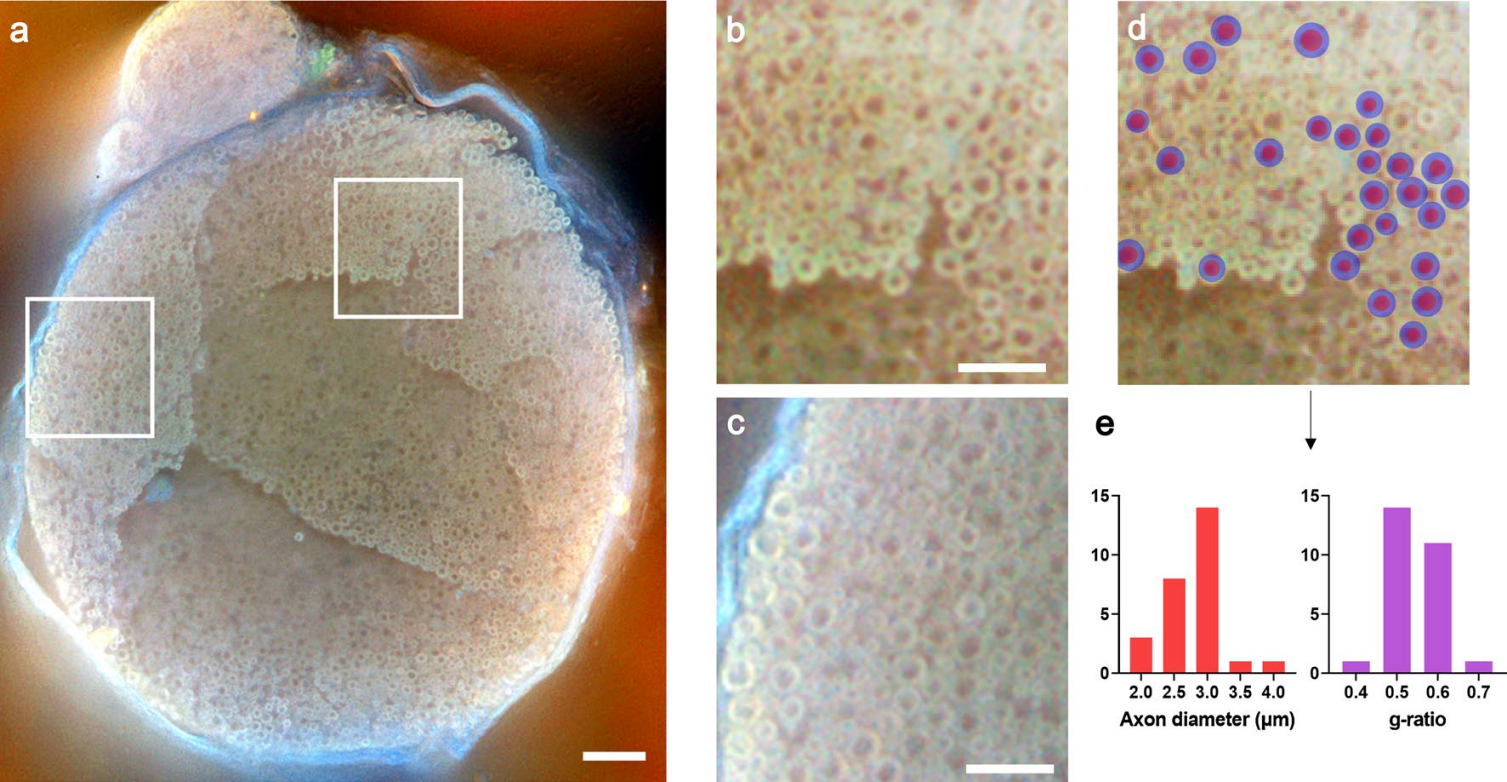
2D-MUSE image of a rat sciatic nerve. Fixed nerve tissue was sectioned on a compresstome and laid immediately on a quartz slide for imaging. Individual myelinated fibers are visible (**b** and **c**) with contrast originating from tissue autofluorescence under UV illumination. (**d**) Manual segmentation on a subset of myelinated fibers in (**b**) allows quantification of axon diameters and corresponding g-ratios, displayed as histograms in (**e**). Scale bars are: (**a**) 100 µm, (**b** and **c**) 50 µm. Close-up views in (**b**) and (**c**) were contrast enhanced to improve visibility of myelin sheaths.

In Fig. 1, we demonstrate 2D-MUSE imaging of a rat sciatic nerve sample, utilizing fixative autofluorescence. Tissue preparation steps included formalin fixation, sectioning with a razor blade, and gentle pressing of the cut tissue onto the quartz slide of the inverted MUSE microscope. When imaging the nerve at this level, we identify three fascicles, separated by thin connective sheaths (perineurium). Image resolution and contrast were found to be sufficient to delineate axons and myelin sheaths of individual nerve fibers. Under the assumption that the space inside the myelin sheath is completely filled with the axon, individual g-ratios can be extracted from these images^29^. The g-ratio relates to the conduction velocity in nerve fibers and is calculated as the ratio of axon diameter to the total fiber diameter. By performing manual segmentation on a group of randomly selected nerve fibers (Fig. 1d), we quantify both axon diameters (2.78 + /−0.41 µm, n = 27), and individual g-ratios (0.55 + /−0.06, n = 27, Fig. 1e).

**Figure 2:**
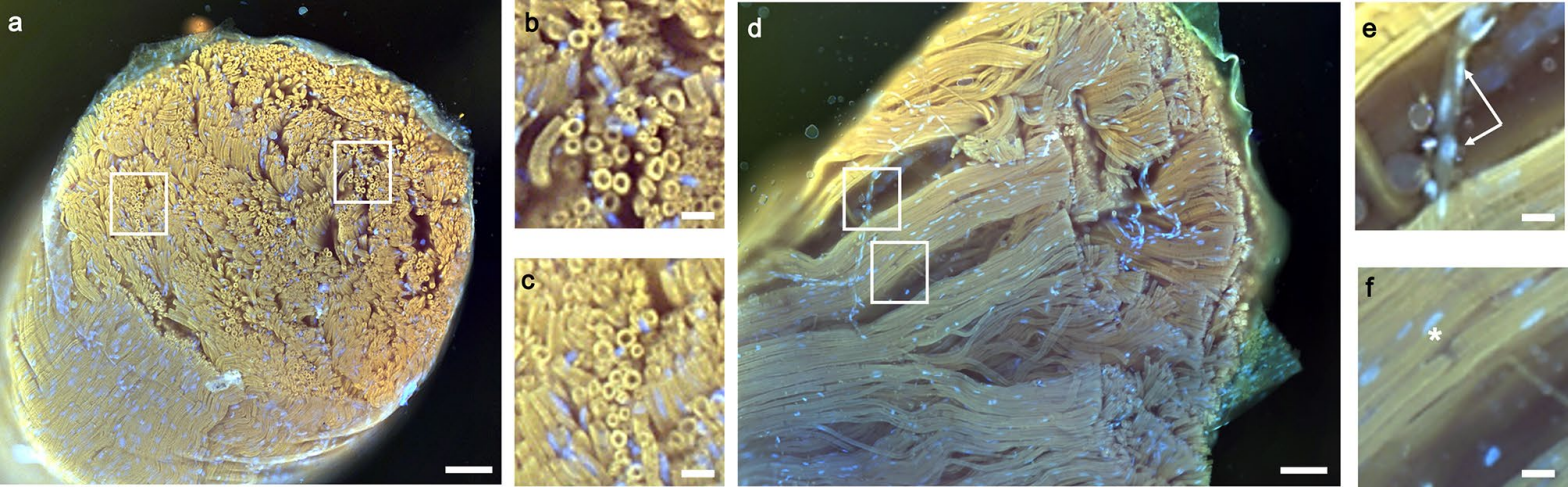
2D-MUSE image of a rat sciatic nerve with staining. Sample was sectioned by hand with a razor blade, immersed in a rhodamine and Hoechst staining solution for 30 s, rinsed in DI water and imaged. (**a**) Transverse cross-section illustrates several myelinated fibers grouped as a single fascicle and surrounded by a thin perineural sheath. (**b** and **c**) Close up views from (**a**) show multiple myelinated fibers as yellow rings. (**d**) Longitudinal cross section allows visualizing fibers along the length of the nerve. (**e**) Zoomed in region from (**d**) illustrates microvasculature revealed by Hoechst staining and evident from tubular structure (arrows). (**f**) A gap in the myelinated fibers is visible in this close-up view, suggestive of Schimdt-Lanterman clefts or node of Ranvier (asterisk). Scale bars are: 100 µm (**a**, **d**) and 20 µm (**b**, **c**, **e**, **f**).

**Figure 3:**
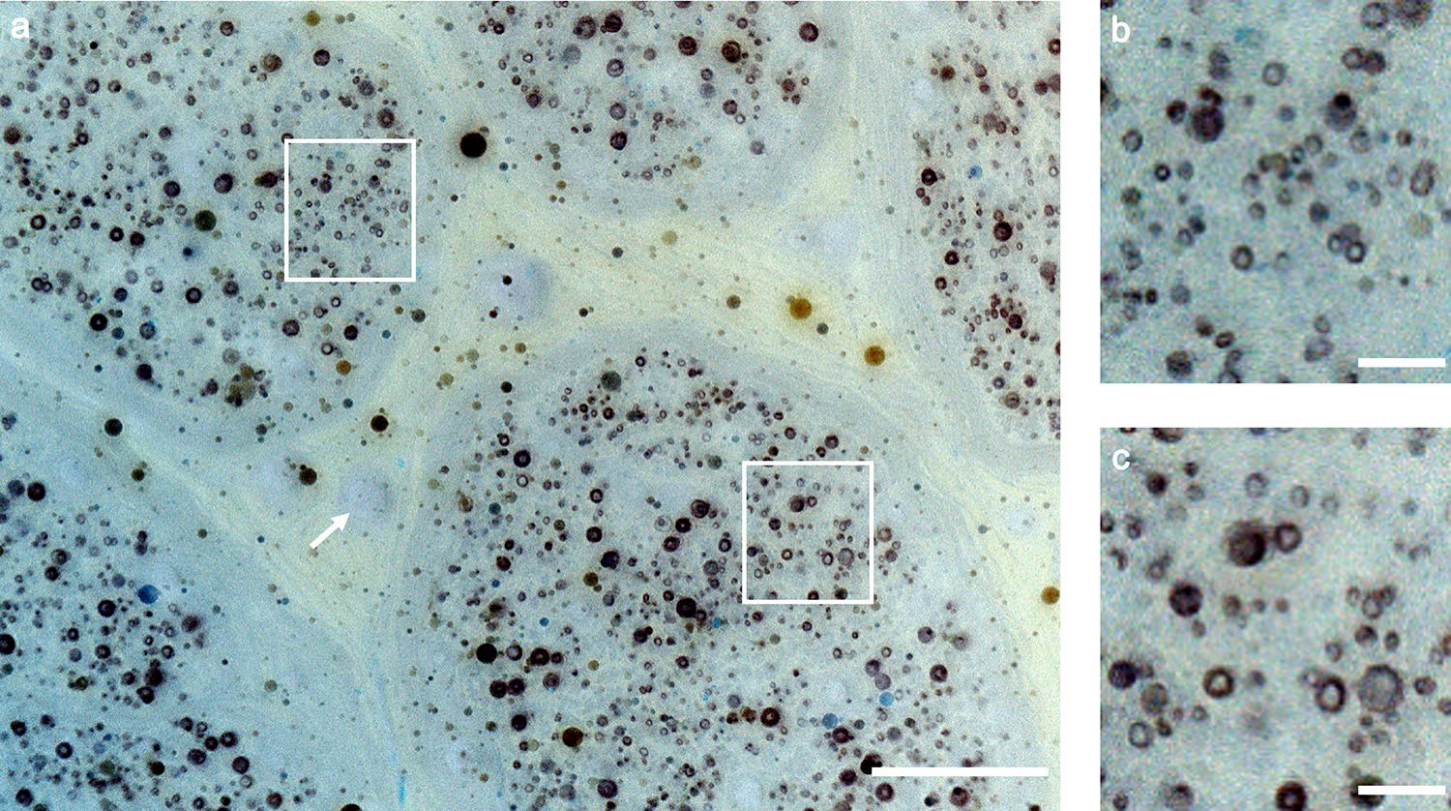
2D-MUSE image of a cadaver tibial nerve sample, stained with a myelin-specific fluorescent compound (Case Imaging Compound^30^). Images were color and intensity inverted to improve visual saliency. (**a**) MUSE image, color and intensity inverted to improve visual saliency. (**b**, **c**) Close up views of regions highlighted by white boxes in (**a**) displaying dark rings, indicative of myelinated fibers. Note the low image contrast from vasculature with this stain (arrow in **a**). Scale bars are 100 µm in (**a**) and 50 µm in (**b** and **c**).

Staining with fluorescent dyes further improved image contrast (Fig. 2). Common MUSE dyes such as Hoechst and rhodamine generated contrast between additional tissue structures, including cell nuclei and vasculature. When imaging the nerve in the longitudinal direction, individual fibers can be clearly delineated from each other across the nerve. Vascular-like structures are evident from Hoechst staining in a close-up view (Fig. 2e). For certain fibers, a gap in myelinated regions is observed, suggestive of Schmidt-Lanterman clefts or nodes of Ranvier. We also tested staining with a fluorescent compound that selectively binds to myelin sheaths (Case Imaging Compound^30^, a stilbenzene derivative). The compound was found to be fluorescent under MUSE illumination, allowing visualization of myelin sheaths on a human tibial nerve (Fig. 3). Note that even with staining, 2D-MUSE imaging is quite fast, with sample preparation and imaging completed in under five minutes.

### Block-face 3D-MUSE imaging

**Figure 4:**
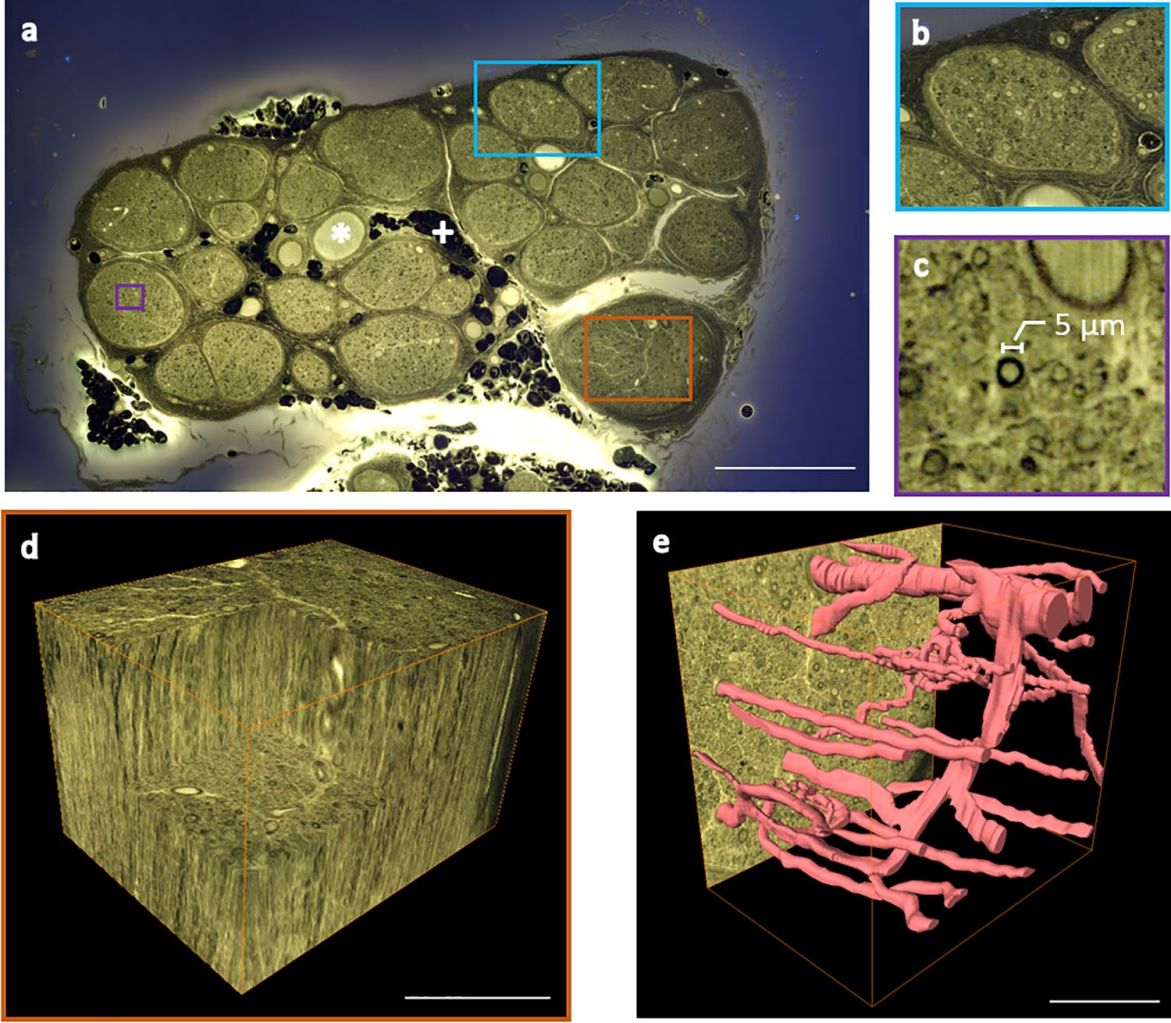
Imaging a cadaver tibial nerve sample with block-face 3D-MUSE imaging. The nerve was post-fixed with osmium tetroxide and counterstained with rhodamine B. Images were acquired with a 10 × 0.45 NA objective and the sample was sectioned at 5 µm thickness. Further details of the system are provided in “Methods”. (**a**) Representative block face image stitched from twelve individual tiles. Structures of interest include fascicles, venuole (asterisk), and adipose cells (plus). (**b** and **c**) Close up views indicate individual fascicles and myelinated fibers respectively. (**d**) Volume rendering of a region within a fascicle, outlined by a brown box in (**a**). (**e**) Manual segmentation of intraneural vasculature from the same volume as in (**d**). Box size: 800 × 600 µm^2^. Scale bars are (**a**) 1 mm (**d** and **e**) 250 µm.

Since 2D-MUSE generated promising images of micro-anatomy in nerve cross-sections, we proceeded to use the block-face, 3D-MUSE imaging system to further reveal spatial organization of peripheral nerves. We used osmium tetroxide and rhodamine B to whole mount stain the nerve, giving contrast to fascicles, connective tissue, myelinated fibers, vasculature, and adipocytes in a human tibial nerve. Individual fascicles along with inner and outer perineural boundaries are visible in the block-face images (Fig. 4a-b). Nerve fibers with diameter greater than 5 µm could be clearly resolved with the current imaging system. An example of a fiber with an axon diameter around 5 µm is illustrated in Fig. 4c. When staining with osmium tetroxide, unsaturated fats in adipocytes react in the same manner as myelin sheaths and therefore, also appear as dark regions in the block-face images. Using a manual segmentation of the inner and outer boundaries of the perineural sheaths, fascicle diameters and perineural sheath thickness could be quantified in the block-face image shown in Fig. 4a. Fascicle diameter was found to be 423 + /− 143 µm and the sheath thickness was 25 + /−11 µm (n = 25 fascicles, Fig. S1). Figure 4d demonstrates a sub-volume with fibers and vasculature within a single nerve fascicle. With manual segmentation, we identify vascular networks within the fascicle (Fig. 4e). Since the volume was sectioned at 5 µm thickness, variations in the shape of structures from one slice to the next were low, allowing determination of structure continuity. Such vascular networks supply nutrients to regions distant from the nerve cell body. When the sub-volume was visualized from the side (XZ plane, longitudinal view), we identify multiple myelinated axons (Supplementary Movie 1). Upon stepping through individual slices in this view, we identify that the full length of the axons is not visible in a single slice but extends over multiple slices. This indicates that axon morphology is not perfectly described by a cylinder and necessitates a more complex 3D description. Volumetric imaging revealed a fascicle merging event (Supplementary Movie 2). The two fascicles are initially seen close to one another and are separated by a perineural sheath. As we move deeper into the nerve, we identify that the sheath gets progressively thinner, until there is merging.

## Discussion

With 2D-MUSE imaging, we identified several key structures in nerve micro-anatomy including myelinated fibers, blood vessels and connective tissue either with auto-fluorescence or by the addition of suitable fluorescent stains. Measurement of axon diameters and g-ratios was possible. The results are similar to previously published values in literature^31^ (mean axon diameter: 2.78 µm vs. 2.28 µm, mean g-ratio: 0.55 vs. 0.52). The minor differences are potentially due to varying tissue expansion or shrinkage during sample preparation. However, since a small group of fibers were randomly selected for our measurements, further tests were not performed due to limited statistical power. Rhodamine B staining improved the visibility of myelin, presumably due to a higher solubility in lipophilic media^32^. The longitudinal cross-section of the nerve showed anatomical structures that are potentially blood vessels and Schmidt-Lanterman clefts and/or nodes of Ranvier, however, further validation is necessary. When staining with the Case Imaging Compound, fluorescence was restricted to the myelinated fibers alone, which could be useful when studying disorders related to the myelin sheath (e.g., multiple sclerosis) and/or assess therapeutic response.

We developed a whole-mount staining approach for visualizing nerve micro-anatomy with 3D-MUSE. Osmium tetroxide preserved lipids/myelin during the dehydration process and produced dark staining for myelinated fibers across depth^33^. An additional advantage of this stain is its role as a contrast agent in micro-CT^34^, suggesting suitability in a multimodality imaging pipeline. Combined with rhodamine B counterstaining, the system provided sufficient resolution and contrast to visualize various structures of interest across depth. For example, nerve fascicles were clearly visible and individual fascicle diameters could be quantified. Volumetric imaging also allowed identification of fascicle merging events. Additionally, inner and outer perineural boundaries can be identified, allowing us to quantify sheath thickness. Since these anatomical parameters (fascicle diameter, sheath thickness, fiber distance from the electrode) significantly affect stimulation response, their measurement can guide computational modeling studies for neuromodulation. With suitable segmentation tools, these datasets can provide an accurate representation of nerve micro-anatomy, making them suitable for modeling studies assessing various nerve interface designs. Visualizing nerve fibers across length illustrated the complex 3D morphology of individual axons, consistent with findings from previous reports utilizing electron microscopy^35^. The combination of osmium tetroxide staining and GMA embedding also produces final tissue blocks with lesser shrinkage compared to paraffin^36^, thereby making the technique more suitable for morphometric analyses. During our embedding experiments, we noticed that the final blocks occasionally contained air bubbles, potentially due to leftover osmium in the tissue interfering with GMA polymerization^37^. We aim to address this limitation in future work by testing optimized protocols prescribed in the literature^38^.

We demonstrated 3D-MUSE block-face imaging and identified advantages of using this system to image peripheral nerves. First, by performing serial section and imaging, long samples can be imaged in a straightforward manner. With the section-and-image approach, the length of nerve imaged could be virtually unlimited. Our current prototype is limited to about 20 mm. Second, since MUSE utilizes simple instrumentation^12^, the system does not require significant technical expertise to develop and maintain. Third, with a tiling approach, a large field of view (up to 20 × 20 mm with our current motorized stages) can be covered. Finally, with our whole-mount staining procedure described above, we generate images of nerve micro-anatomy with improved resolution and contrast as compared to micro-CT and MRI techniques. For instance, micro-CT imaging provides limited contrast to visualize the perineural sheaths around the fascicles. Diffusion MRI provides maps of fiber tracts but is limited in resolution and contrast to visualize individual fibers. We identify that with our 3D-MUSE approach, both perineural sheaths and large nerve fibers are visible.

Overall, our work describes a microscopic imaging method based on MUSE imaging to visualize nerve micro-anatomy in 2D and 3D. The 2D imaging system offers a simple method to image myelinated fibers at specific locations in the nerve ex vivo, without the need for laborious embedding protocols or complex instrumentation. Extending MUSE to 3D via block-face imaging offers a simple, relatively inexpensive method to analyze these samples when a continuous 3D representation of nerve anatomy is required.

## Methods

### Tissue preparation

Short segments of sciatic nerves were excised from discarded rat tissues from unrelated studies with IACUC approval at Washington University in St. Louis. Results presented in this work are in accordance with relevant guidelines and regulations (ARRIVE guidelines^39^). Nerves were fixed in 4% PFA overnight and were subsequently rinsed and stored in 1x PBS. Samples were either cut by hand with a razor blade or were embedded in agarose and sectioned on a Compresstome® (Precisionary, MA) to create a flat surface. Human tibial nerve samples were extracted from embalmed cadavers after their use in medical school cadaver lab training. The study was conducted according to guidelines and regulations laid down in the Declaration of Helsinki. Informed consent was provided by the donors prior to their deaths or by their next of kin. Our study was reviewed by the Case Western Reserve University Institutional Review Board and was determined as non-human subject research based on the nature of the de-identified cadaver donations and other study parameters. Samples were stored in 10% neutral buffered formalin solution at 4 °C for several weeks prior to staining and embedding. Nerves were cut into < 1 mm sections with a razor blade and washed in 1x PBS overnight at 4°C to remove excess fixative.

### Sample preparation for 2D-MUSE imaging

Rat sciatic nerve samples were immersed in a solution of 0.5 mg/mL rhodamine B (Sigma) and 0.1 mg/mL Hoechst (Sigma) in 1x PBS for approximately 30 seconds, followed by rinsing under DI water for 10 seconds. Human tibial nerve samples were stained with a myelin-specific fluorescent compound (Case Imaging Compound^30^). The compound was prepared and stored at a concentration of 10 mM in DMSO. Prior to imaging, the staining solution was made by diluting to a final concentration of 1 mM with 1x PBS. Steps involved in this sample preparation protocol are illustrated in Fig. S2a.

### Sample preparation for block-face 3D-MUSE imaging

Human tibial nerve samples were stained with osmium tetroxide and rhodamine B followed by embedding in glycol methacrylate (GMA) resin. The samples were transferred to 2% osmium tetroxide (Electron Microscopy Sciences) and placed on a rotator for 2 hours, forming a black, insoluble precipitate upon reaction with unsaturated fatty acids within the tissue^33^. The sample was dehydrated in a series of ethanol solutions (30, 60, 90 and 100%, 2h each). Rhodamine B was added at a concentration of 0.5% (w/v) to the ethanol solutions to create a counter contrast. The dehydrated sample was embedded in the GMA resin as previously described^40^. The samples were placed in a 50:50 mix of ethanol and GMA monomer (Electron Microscopy Sciences) overnight at 4 °C, followed by a freshly prepared GMA infiltration solution (GMA monomer with 1% (w/v) of the initiator added) for 24 h at 4 °C. Samples were then polymerized in 1:15 mix of accelerator and infiltration solution in Teflon molds under vacuum overnight at 20–25 °C. Hardened sample blocks were attached to plastic holders using a fast-curing methyl methacrylate resin (Electron Microscopy Sciences) for microtome sectioning. A detailed protocol is illustrated in Fig. S2b.

### 2D-MUSE imaging

The setup for 2D-MUSE imaging consists of a UV transparent slide (commonly quartz), 280 nm UV LED for illumination and an inverted microscope system. After the samples were prepared as described above, they were placed on the slide with the surface to be imaged facing downward. The slide is attached to a motorized XYZ stage to provide automated panning and focusing of the sample surface. Since UV light at 280 nm is absorbed by glass, the sample surface is excited with oblique illumination. This is in contrast to routine epifluorescence microscopes which use light in the visible wavelength for excitation with coaxial illumination. The microscope system included a 10x/0.45 NA objective (Nikon Plan Apo), an infinity corrected tube lens (ITL200, Thorlabs) and a CCD color camera (Ximea, MD091CU-SY). Custom control software integrating the various hardware components was developed in the. NET framework. The imaging protocol consists of focusing the microscope at a specific region of interest and capturing an image. Optionally, tiling can be performed to increase the effective field of view. Further details about the imaging system are available in a previous publication by Fereidouni et al.^12^

### Block-face 3D-MUSE imaging

The setup for block-face 3D-MUSE imaging system is based on an automated microtome and a MUSE imaging system. Serial sectioning was performed at 5 µm section thickness with a motorized rotary microtome (Microm HM355 S) equipped with a 16 cm D profile tungsten carbide knife. The GMA-embedded sample was trimmed repeatedly with the microtome. At the end of each cutting cycle, the microtome positions the sample carrier at its upper reversal point (away from the knife) making it convenient to perform serial imaging. An Arduino microcontroller and relay switches were used as the interface between the computer and the microtome. The MUSE imaging system was similar to the original MUSE setup (Fig. S3a)^12^. For illumination, we use a UV LED with a center wavelength of 285 nm (Thorlabs). Images were collected using a 10x/0.45 objective (Nikon Plan Apo) and a CMOS color camera (Teledyne FLIR, 12 megapixels), providing a field of view of about 1.4 x 1.1 mm. The imaging system is mounted on 3-axis motorized stages (Zaber) to facilitate focusing and tiling. Micro-Manager^41^, an open-source microscope control software, was used to control serial sample sectioning and image acquisition. Image tiles were set up with an overlap of ten percent to ensure stitching was free of artifacts. A resolution test image of the USAF 1951 target taken with this system is provided in Fig. S3b, indicating resolvability up to Group 9 Element 2 (0.87 µm line spacing).

### Image post-processing and visualization

Raw MUSE data were post-processed with the procedure described previously^12^. Briefly, images were sharpened using unsharp masking in MATLAB. Image contrast was enhanced either with the automatic white balance tool or the curve tool in GIMP. Tile scans were stitched using Image Composite Editor software (Microsoft).

### Segmentation and quantification methods

Manual segmentation tools were used to create labeled images for subsequent quantitative analyses. Structures were identified by visually comparing our results with the gold standard of resin embedding and toluidine blue staining^42^. In order to compute fiber diameters and g-ratios (Fig. 1d and e), large fibers were randomly selected and the inner and outer boundaries of myelin sheaths were delineated using the free hand segmentation tool in Amira software (Thermo Fisher Scientific). A subset of myelinated fibers with diameters ≥ 4 µm could be identified and labeled with this approach. Smaller fibers were difficult to accurately label due to limitations in image resolution. The labeled regions were subsequently analyzed by computing the diameter of a circle with the same area as the region. This was done using the regionprops command in MATLAB. Since the command returns the diameter in terms of pixels, values were multiplied by the calibrated pixel size to obtain the result in physical units (µm).

A similar approach was taken to quantify fascicle diameters and perineural sheath thickness. The inner and outer boundaries of the perineural sheath were delineated to create a labeled image (shown overlaid against the raw image in Fig. S1). We then computed equivalent fascicle diameters. Additionally, perineural sheath thickness was determined by measuring the thickness at 100 points along its boundary using MATLAB code developed in-house. These points were created by drawing rays from the fascicle centroid at equiangular spacing, thereby covering a full 360 degree rotation. This process was repeated for each of the 25 fascicles in Fig. 4a. All values presented in this work indicate mean + /−SD.

### Ethical approval

All animal procedures and protocols were performed in accordance with relevant guidelines and regulations (NIH Guide for the Care and Use of Laboratory Animals).

## Supplementary Information

Supplementary Information 1.

Supplementary Information 2.

Supplementary Video 1.

Supplementary Video 2.
