# Supplementary material for: Imaging peripheral nerve micro-anatomy with MUSE, 2D and 3D approaches
Source: Sci Rep. 2022 Jun 17;12:10205. doi: 10.1038/s41598-022-14166-1 (PMC9205958; doi:10.1038/s41598-022-14166-1)
Supplement: Supplementary file 1 — Supplementary Information 1. [file 41598_2022_14166_MOESM1_ESM.docx]

**Description of Additional Supplementary Files**

File Name: Supplementary Video 1

Description: Flythrough videos of a human tibial nerve sample, stained with osmium tetroxide and rhodamine B and imaged with a section thickness of 5 µm (voxel size: 0.37 x 0.37 x 5 µm). A total of 90 2D slices were used to create this 3D volume, rendered in Amira. Volume was up-sampled in the z direction (5x) to create these flythrough stacks in XY and XZ. The nerve was sectioned in a transverse manner, such that the nerve cross-section is visible in each section.

File Name: Supplementary Video 2

Description: Flythrough video of a human tibial nerve sample, stained with osmium tetroxide and rhodamine B and imaged with a 5x 0.14 NA objective and a section thickness of 3 µm (voxel size: 0.74 x 0.74 x 3 µm). A fascicle merging event is visible, with a gradual thinning of the perineural sheath separating the two fascicles. Manual segmentations of fascicle regions are converted to create a surface rendering.
