# Supplementary material for: Imaging peripheral nerve micro-anatomy with MUSE, 2D and 3D approaches
Source: Sci Rep. 2022 Jun 17;12:10205. doi: 10.1038/s41598-022-14166-1 (PMC9205958; doi:10.1038/s41598-022-14166-1)
Supplement: Supplementary file 2 — Supplementary Information 2. [file 41598_2022_14166_MOESM2_ESM.docx]

**Supplementary figures**

**Imaging peripheral nerve micro-anatomy with MUSE, 2D and 3D approaches**

Chaitanya Kolluru^1,+^, Austin Todd^2,+^, Aniruddha R. Upadhye^1,3^, Yehe Liu^1^, Mikhail Y.Berezin^4^, Farzad Fereidouni^5^, Richard M. Levenson^5^, Yanming Wang^6^, Andrew J. Shoffstall^1,3^, Michael W.Jenkins^1,7^, and David L. Wilson^1,6^

^1^Department of Biomedical Engineering, Case Western Reserve University, Cleveland, OH, 44106, USA

^2^University of Texas Health Science Center at San Antonio, San Antonio, TX, 78229, USA

^3^APT Center, Louis Stokes Cleveland VA Medical Center, Cleveland, OH, 44106, USA

^4^Department of Radiology, Washington University in St. Louis, St. Louis, MO, 63110, USA

^5^Department of Pathology and Laboratory Medicine, UC Davis Health, Sacramento, CA, 95817, USA

^6^Department of Radiology, Case Western Reserve University, Cleveland, OH, 44106, USA

^7^Department of Pediatrics, Case Western Reserve University, Cleveland, OH, 44106, USA

^*^[dlw@case.edu](mailto:dlw@case.edu)

^+^Both authors contributed equally to this work.


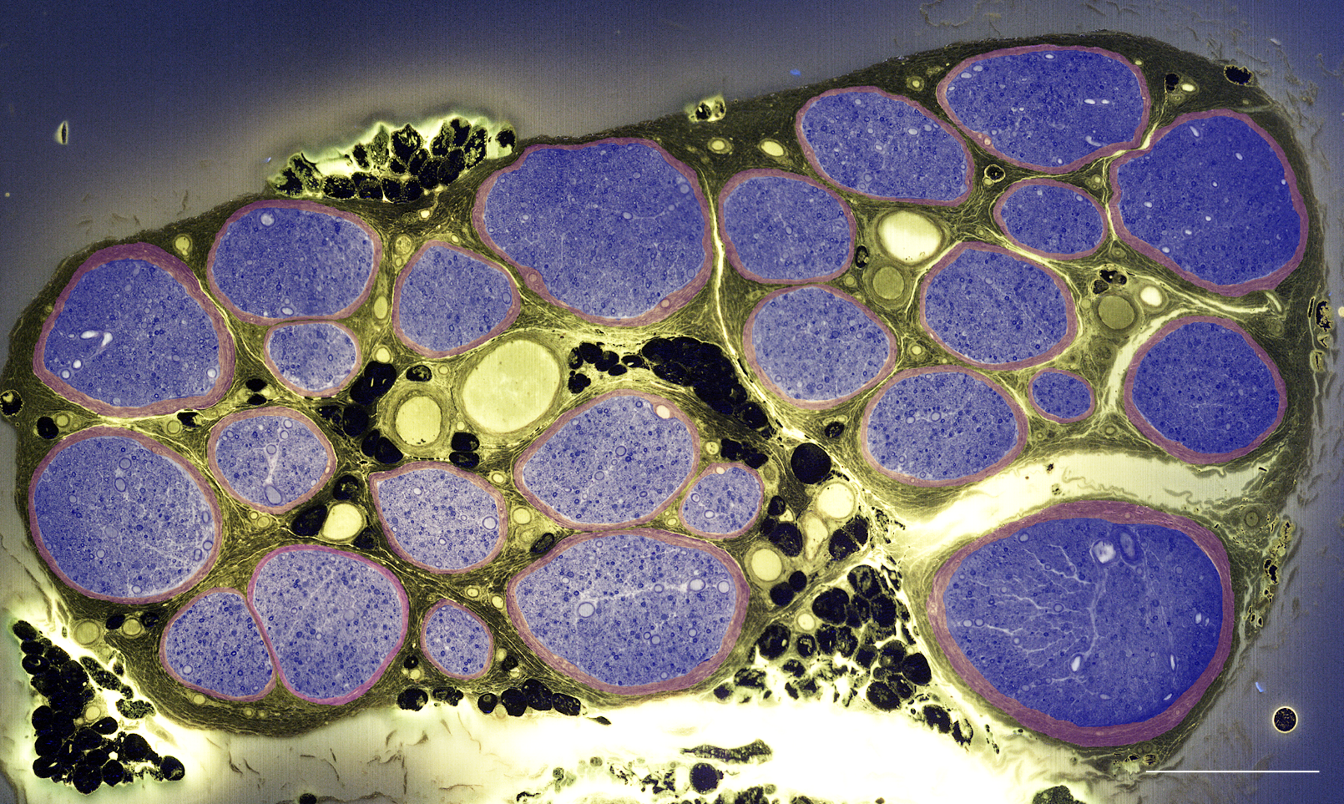


Figure S1. Manual segmentation of the perineurium overlaid with the MUSE block-face image. Fascicles are highlighted in blue and perineurium is displayed in red. Scale bar: 1 mm.


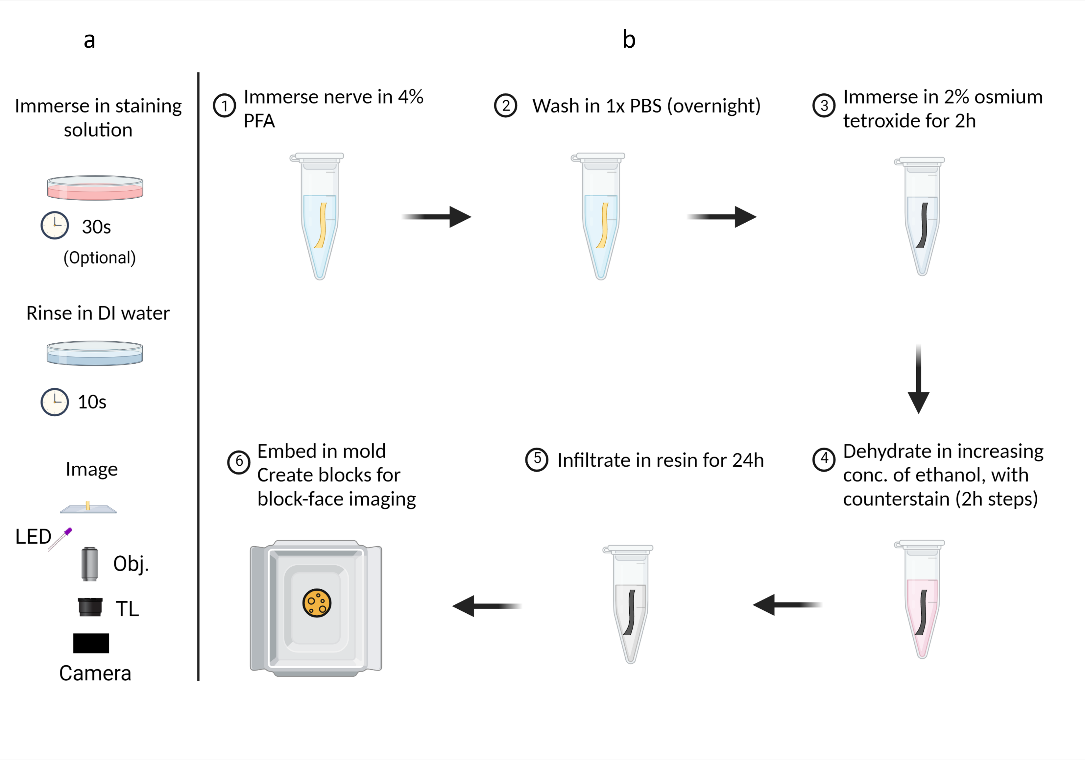


Figure S2. Schematic of the sample preparation protocols for imaging nerves with 2D-MUSE (left), and block-face 3D-MUSE imaging (right). Obj: Objective, TL: Tube lens. Image created with [biorender.com](http://biorender.com).


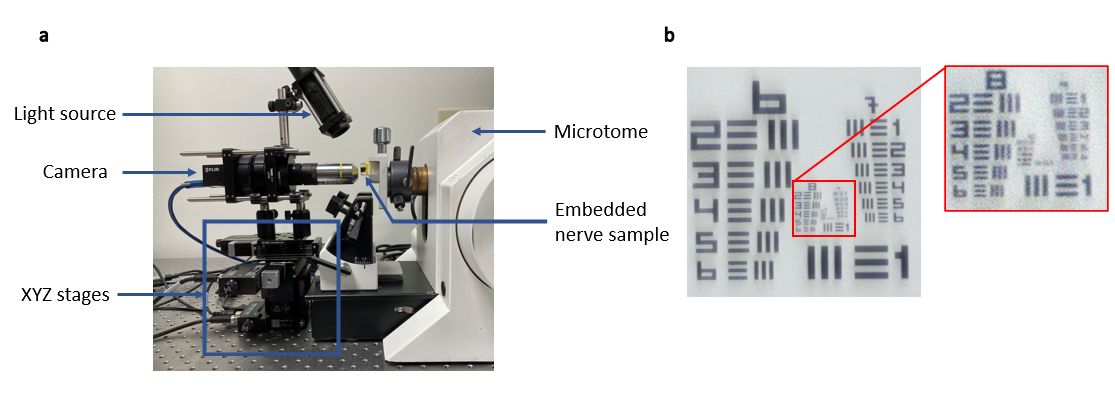


Figure S3. (a) Components in the block-face 3D-MUSE imaging system. (b) Measuring image resolution of our system with a USAF 1951 test target^1^. Resolvability up Group 9 Element 2 (0.87 µm line spacing) can be achieved.

**References**

1. Sun, H. Geometrical Optics. in *Basic Optical Engineering for Engineers and Scientists* vol. PM294 41–42 (SPIE, 2019).
